# Low-pass shotgun sequencing of the barley genome facilitates rapid identification of genes, conserved non-coding sequences and novel repeats

**DOI:** 10.1186/1471-2164-9-518

**Published:** 2008-10-31

**Authors:** Thomas Wicker, Apurva Narechania, Francois Sabot, Joshua Stein, Giang TH Vu, Andreas Graner, Doreen Ware, Nils Stein

**Affiliations:** 1 Institute of Plant Biology, University Zurich, Zollikerstrasse 107, 8008 Zurich, Switzerland; 2 Cold Spring Harbor Laboratory, 1 Bungtown Rd., Cold Spring Harbor, NY 11724, USA; 3 Laboratoire Génome et Développement des Plantes, UMR 5096 CNRS-IRD-Université de Perpignan, 52 Avenue Paul Alduy, F-66860 Perpignan, France; 4 United States Department of Agriculture-Agricultural Research Service (USDA-ARS) North Atlantic Area (NAA) Plant, Soil & Nutrition Laboratory Research Unit, Ithaca, New York 15853, USA; 5 Leibniz Institute of Plant Genetics and Crop Plant Research (IPK), Corrensstr. 3, 06466 Gatersleben, Germany; 6 Institute of Biological, Environmental and Rural Sciences (IBERS), Edward Llwyd Buiding, Aberystwyth University, Ceredigion, SY23 3DA, UK

## Abstract

**Background:**

Barley has one of the largest and most complex genomes of all economically important food crops. The rise of new short read sequencing technologies such as Illumina/Solexa permits such large genomes to be effectively sampled at relatively low cost. Based on the corresponding sequence reads a Mathematically Defined Repeat (MDR) index can be generated to map repetitive regions in genomic sequences.

**Results:**

We have generated 574 Mbp of Illumina/Solexa sequences from barley total genomic DNA, representing about 10% of a genome equivalent. From these sequences we generated an MDR index which was then used to identify and mark repetitive regions in the barley genome. Comparison of the MDR plots with expert repeat annotation drawing on the information already available for known repetitive elements revealed a significant correspondence between the two methods. MDR-based annotation allowed for the identification of dozens of novel repeat sequences, though, which were not recognised by hand-annotation. The MDR data was also used to identify gene-containing regions by masking of repetitive sequences in eight de-novo sequenced bacterial artificial chromosome (BAC) clones. For half of the identified candidate gene islands indeed gene sequences could be identified. MDR data were only of limited use, when mapped on genomic sequences from the closely related species *Triticum monococcum *as only a fraction of the repetitive sequences was recognised.

**Conclusion:**

An MDR index for barley, which was obtained by whole-genome Illumina/Solexa sequencing, proved as efficient in repeat identification as manual expert annotation. Circumventing the labour-intensive step of producing a specific repeat library for expert annotation, an MDR index provides an elegant and efficient resource for the identification of repetitive and low-copy (i.e. potentially gene-containing sequences) regions in uncharacterised genomic sequences. The restriction that a particular MDR index can not be used across species is outweighed by the low costs of Illumina/Solexa sequencing which makes any chosen genome accessible for whole-genome sequence sampling.

## Background

Within the family of *Poaceae*, the tribe of the Triticeae contains some of the world's most important food crops such as wheat and barley. Despite their economic importance, efforts to produce large amounts of genomic DNA sequences have been dragging along slowly due to the enormous complexity and the high repeat content of the Triticeae genomes. Barley, for example, has a genome size of 5,500 Mbp [1], almost twice the size of the human genome, which is composed of over 80% of repetitive DNA [2]. Transposable elements (TEs) make the major part of the repetitive fraction, some TE families can reach thousands of copies. The most abundant TE family, *BARE*, is e.g. present in 80'000 to 120'000 copies, contributing about 9.6% to the total genomic sequence [3-5].

Such vast amounts of repetitive DNA hinder the efficient large scale sequencing and also make sequence analysis much more complicated and time-consuming. Identification and characterisation of transposable elements have, therefore, been an important aspects of Triticeae genomics from the beginning, and the public database TREP was dedicated to host and document annotated repeat sequences from Triticeae [6].

Triticeae researchers are facing a double challenge in genome sequencing. First, an adequate amount of genomic sequences needs to be produced to reasonably cover these huge genomes and second, at the same time methods to efficiently analyse the accumulated large amount of data need to be developed. While new second generation sequencing technologies such as Illumina/Solexa 1G, Roche/454 GS FLX or ABI SOLiD provide the opportunity to produce genomic sequences at ever-increasing speed and decreasing costs [7], this step change in technology entails the need for efficient tools and strategies to rapidly narrow down and to accurately describe DNA regions of interest in an ocean of unorganised sequence data.

Mathematically defined repeats (MDRs) are determined by measuring the frequency of k-mer (e.g. a 20-nucleotide oligomer) matches within a sequence dataset. MDRs offer the advantage of requiring no prior knowledge of structurally-defined repetitive elements to identify repeats within a genome. They have been previously used to aid genome assembly by masking repetitive sequences [8,9], to identify TEs [10] and reconstruct ancestral TE families [11]. However the full capabilities of MDR analysis can be difficult to achieve due to the high memory requirements of previously implemented data structures (e.g[11]). The accompanying article [12] introduced a highly efficient tool, Tallymer, to analyze k-mers within the massive sequence datasets available from maize. Tallymer uses an enhanced suffix array data structure that offers reduced hard disk space requirements and processing times compared to suffix trees, permitting quick processing of very large sequence sets.

Repeat masking is an efficient way to narrow down the amount of sequences that may contain genes. This process basically searches the sequence of interest for and flags all regions that show homology to known TEs and other repetitive sequences. The bases in the repeat regions are then replaced either by "X" or "N" (hard masking) or lowercase letters (soft masking). Examples for publicly available repeat masking software are RepeatMasker [13] or CENSOR [14]. Obviously, the repeat masking process is only as complete as the repeat database it is based upon. Low-copy repeats and variable regions within TE families are usually not recognised.

In this study, we present a practical application of short-read sequencing to the large and complex genome of barley. We raised in a single run of Illumina/Solexa 1G sequencing almost 10% of the sequence information present in the haploid barley genome equivalent. The raw sequences were used to generate an MDR index and mapped to publicly available genomic sequences in order to identify repetitive and low-copy sequences. The so-called MDR plots allowed an efficient exclusion of repetitive sequences and easy identification of gene-containing space. The MDR plots were compared with results of careful expert re-annotations of the same sequences, showing a large overlap but also the complementary value of both approaches. It demonstrated that expert annotation of Triticeae repetitive DNA has already reached a high level of accuracy based on a very limited amount of available Triticeae genomic sequences but the MDR data still led to the identification of several, novel TE sequences.

## Methods

### DNA sequencing – Sanger BAC DNA sequencing

Eight barley bacterial artificial chromosome (BAC) clones originating from the library of cultivar Morex [15] were sent to commercial Sanger shotgun sequencing (AGOWA, Berlin, Germany). Each clone was sequenced at two- to three-fold coverage considering an average BAC insert size of 110 kb. Sequence data was further processed by the program PHRED [16] and assembled using the program GAP4 (Roger Staden, MRC Cambridge). Contig consensus sequences were exported into FASTA-flatfiles sorted according to contig length without considering their true physical order in the respective BACs.

### DNA sequencing – high-throughput short read shotgun sequencing

Genomic DNA was extracted from barley cultivar Morex as previously described [17]. The DNA was adjusted to a concentration of 1 μg/μl and sent for commercial short read sequencing on the Illumina Genome Analyzer platform (GATC Biotech, Konstanz, Germany). Genomic DNA was fragmented and a size fraction of around 300 bp was selected for sequence sample preparation according to manufacturer's instructions. 15,950,203 individual reads each of 36 nt in length were exported out of the raw data file obtained from 6 lanes of a flowcell in a single sequencing run by applying the default system settings for quality check. This amounted to 574 Mb whole genome shotgun sequence information. No additional measures of quality clipping were applied before utilising the dataset for MDR index construction.

### Generation of an MDR index and analysis of BAC DNA sequence

All 15,950,203 individual reads were used for construction of the barley MDR index as an enhanced suffix array [12,18,19] of 20-mers, a data structure that allows for fast and efficient retrieval of copy numbers in the index relative to oligomer queries. Algorithms and scripts were developed for use on a Dual 3.8 Ghz Intel 64 bit linux machine with 8GB of memory. In this study, the analysed BAC sequences were virtually digested into overlapping 20-mers and each 20-mer was assessed for its frequency in the index. The resulting data was transformed to reflect the average copy number at each nucleotide position across the BAC sequence. When plotted logarithmically on a genomic scale, these discrete statistics aggregate into a repeat landscape wherein regions of high TE content are easily distinguished from low-copy genic and regulatory content. Indices of this type then become a persistent resource against which query sequences of the same species can be assessed for their repeat content. No special provisions were made to address homopolymers and simple sequence repeats SSRs. The idea was to use available sequence resources directly without any manual or computational annotations/modifications. Thus, homopolymers and SSRs cause spikes of apparently extremely high copy numbers in the MDR plots. These signals are very distinct and can easily be distinguished from high-copy signals that originate from repeated sequences with higher complexity. All software for users to generate and query their own MDR indices is available upon request.

### Expert annotation of publicly available sequences

In order to provide a homogeneous set of annotations for comparisons, we chose a set of publicly available sequences from barley and the diploid wheat *Triticum monococcum *(Table 1 and 2). For each of the two species the cumulative length of the chosen sequences is approximately 2 Mbp. The BAC sequences were re-annotated for both protein coding and TE content. Expert annotation of TEs was done by first identifying TEs by BLASTN [20] against the Triticeae repeat database (TREP,[21]) and hand annotating them afterwards with a visual alignment program DOTTER [22] or Artemis [23]. *De novo *TE detection was performed by BLASTX [20] against PTREP, the protein division of TREP and against all rice proteins. Novel non-coding repeats were detected by BLASTN against a set of 315 publicly available large genomic (mostly BAC) sequences from Triticeae (see Additional file 1). Alternatively, novel TE families were identified using DOTTER based on structural characteristics such as long terminal repeats (LTRs), terminal inverted repeats (TIRs) and target site duplications. For comparative studies and hand annotation of genes, the datasets from the TIGR [24] rice (version 5) and *Arabidopsis *(version 6) genomes were used. Genes were identified by BLASTX against all rice and *Arabidopsis *proteins and annotated by hand comparison of the Triticeae sequence with annotated CDS from rice or *Arabidopsis *or both.

**Table 1:** Previously published barley sequences used for re-annotation.

sequence	size (bp)	genes*	TE content (bp)	Reference
AF474373	124052	9	70585 (56.9%)	[49]
AF521177	211664	14	80220 (37.9%)	[50]
AY268139	120562	2	86442 (71.7%)	[51]
AY485643	114996	10	51173 (44.5%)	[52]
AY642926	184425	5	88708 (48.1%)	[32]
AY643842S2	129099	6	92563 (71.7%)	[53]
AY643842S3	160856	5	128684 (80.0%)	[53]
AY661558	439775	2	385242 (87.6%)	[54]
EF067844	518343	5	422967 (81.6%)	[55]

Total	2003772		1406590 (70.2%)	

*Based on expert annotation; count includes gene fragments and pseudogenes and may differ from gene number given in the respective publication.

**Table 2:** Previously published sequences from diploid wheat T. monococcum used for re-annotation and comparison with their MDR profiles

sequence	size (bp)	genes*	TE content (bp)	Reference
AY491681	101082	8	43970 (43.5%)	[56]
AY951944	190450	4	134076 (70.4%)	[57]
AF459639	215222	5	157972 (73.4%)	[38]
AF326781	211009	5	160577 (76.1%)	[39]
AY146588	285425	4	232906 (81.6%)	[45]
AY485644	438809	8	313748 (71.5%)	[52]
AY188331	133606	1	112229 (84.0%)	[58]
AY188332	95522	2	78041 (81.7%)	[58]
AY188333	112309	1	85242 (75.9%)	[58]

Total	1783434		1318766 (73.9%)	

*Based on expert annotation; count includes gene fragments and pseudogenes and may differ from gene number given in the respective publication.

### Automated repeat masking and processing of MDR data

For repeat masking, the BAC sequences were used in BLASTN searches against the complete TREP database (release 9) which contains multiple individual copies of most TE families and thus covers variable regions within TEs better than a database which contains merely consensus sequences. Additionally, a BLASTX search was done against PTREP in order to identify coding regions of divergent TE families. Regions which produced significant BLAST (E-value < 10e-10) hits were replaced with stretches of "X". This repeat masking was performed with a custom Perl program. The results of this procedure are virtually identical with those of RepeatMasker [13]. We used the described approach rather than RepeatMasker because of its better integration into our custom software package. All parts of TE sequences, protein coding and-non-coding, were masked.

For automatic extraction of novel repeats, regions extending over at least 80 bp with an MDR signal strength of at least 2 were identified. This corresponds to a copy number of about 20, as the Illumina/Solexa dataset covers approximately 10% of a genome equivalent. Since many TEs contain variable regions that would have lower MDR signals, we allowed for stretches of 20 bp for the signal to go below the threshold of 2 (i.e. highly variable or fast-evolving regions within a repeat may have a lower or no coverage at all in the MDR set). For the extraction of MDR data for specific TEs, the position information from the manual annotation was used to obtain the corresponding coverage data from the MDR datafile. In this way, we obtained coverage data for all annotated TEs.

For graphical display of MDR plots and expert annotations, and for processing the MDR and annotation data, custom programs were written in Perl. The source codes of all custom Perl programs that were used for this study are available upon request.

### Sequence accession numbers

The barley genomic Illumina/Solexa reads are available at NCBI's Short Read Archive (accession number SRA001155) or as download at . The unfinished sequences of the eight barley BAC clones are deposited at NCBI Genbank under the accession numbers EU914123 – EU914130.

## Results

### An index of mathematically defined repeats (MDR) for the barley genome

**Figure 1:**
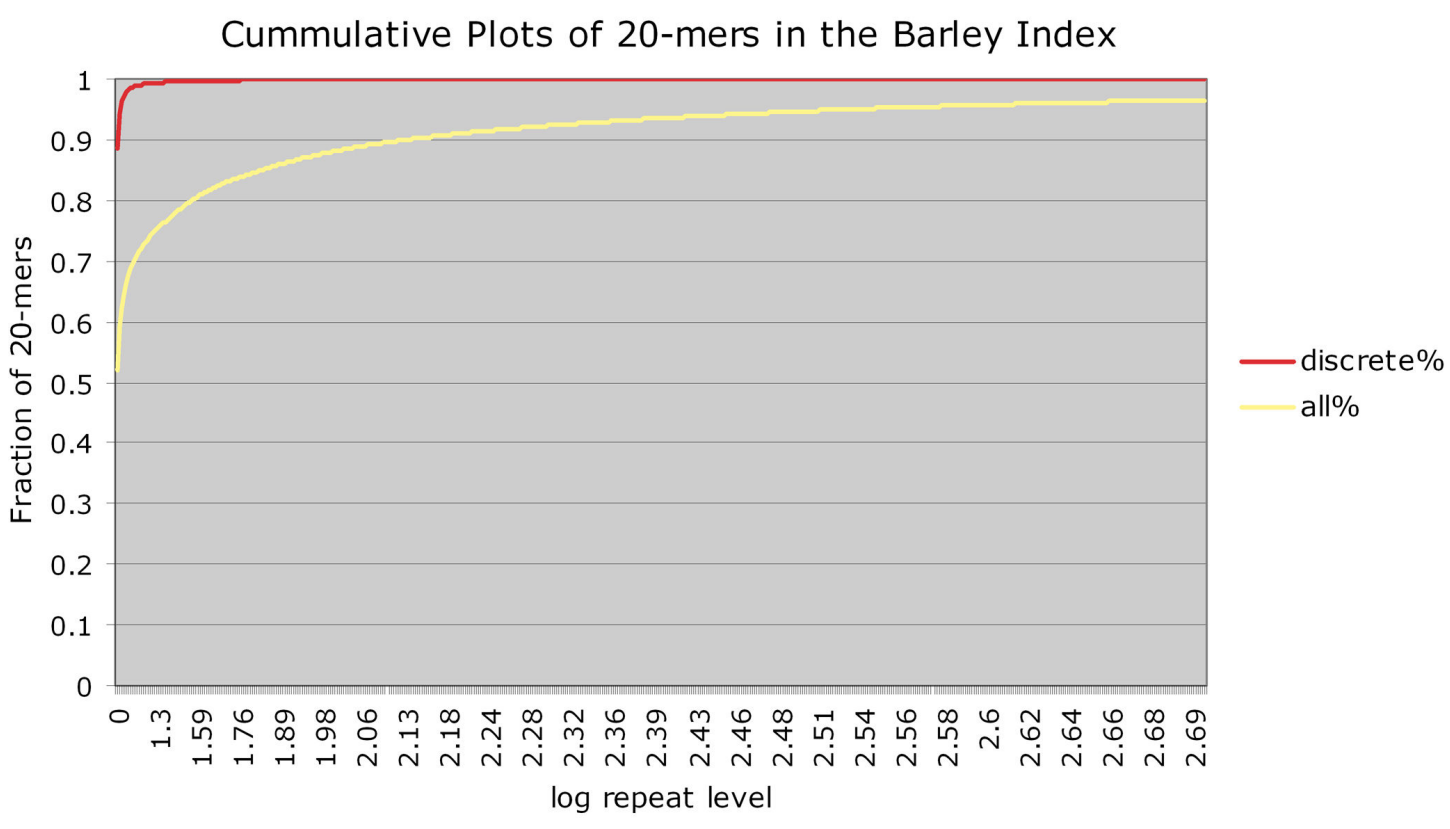
**K-mer composition of the MDR index.** The fraction of collapsed discrete and all 20-mers in the set is shown as a function of the repeat level up to 500 copies. The curve for the collapsed discrete 20-mers converges to 1 rapidly, indicating that most 20-mers in the set are relatively infrequent in the genome. The curve that plots all available 20 mers converges more slowly and is a reflection of a small fraction of high frequency 20-mers in the set.

Over 10% of the barley genome was sampled in the context of a whole genome shotgun (WGS) sequencing experiment, yielding 15,950,203 short reads (each 36 nt in length) and 574,020,163 total nucleotides (574 Mbp). The reads were scanned to identify all 20 mer sequences, generating an index of a total of 269,549,167 20-mers. This set contained 158,770,429 discrete 20 mer sequences. Figure 1 shows the distribution of 20-mers across multiple repeat levels. Distributions of frequency are shown in two ways: (1) discrete 20 mer sequences, and (2) all 20 mers in the set. The former case tabulates how many times each 20 mer sequence is found within the entire set. Of the 158,770,429 discrete 20 mers sequences, 140,444,817 are found only once in the entire set. The most highly represented 20 mer sequence was present 169,559 times. The curve with all 20 mers plots fractions relative to the total number of 20 mers in the set. For example, nearly 99% of discrete 20 mer sequences in the set occur 1–10 times, but this fraction consists of only 71% of all 20-mer occurences in the set. Therefore, given that the solexa library is a representative, unbiased reflection of the barley genome, nearly 30% of its sequence (20-mers that exists 11 or more times) is derived from a mere 1% of discrete 20 mers sequences available.

Chloroplast and mitochondrial DNA were present in moderate amounts in the DNA sample. An MDR plot of the barley chloroplast genome (GenBank accesion EF115541) showed the chloroplast to be evenly covered approximately 60-fold (see Additional file 2). Given a size of 136.4 kb for the cpDNA molecule of barley the genomic DNA sample utilised for WGS sequencing contained a 1.3% fraction of cpDNA. Mitochondrial DNA was less abundant: judging from a short mitochondrial insertion into nuclear DNA (see below) the mitochondrial genome is covered only about 3-fold.

### Comparison of MDR plots with manual annotation

We selected 9 publicly available large genomic sequences from barley with a cumulative length of over 2 Mbp and re-annotated them in detail in order to obtain a standard set of homogeneously annotated sequences. Special attention was given to the analysis of TEs, in order to cover the repetitive fraction as thoroughly as possible (see methods). The 9 sequences range in size from 114 kb to 519 kb (Table 1) and contain 2 to 14 genes, respectively; gene fragments and pseudogenes were included in this count. In some cases, the published annotations contained TE sequences that were mistakenly annotated as genes. Thus, the number of genes we annotated may differ from the ones given in the respective publications. The TE content of the nine barley sequences varied strongly from 37.9% to 87.6% (Table 1). We will hereafter refer to these sequences as the "standard sequence set".

**Figure 2:**
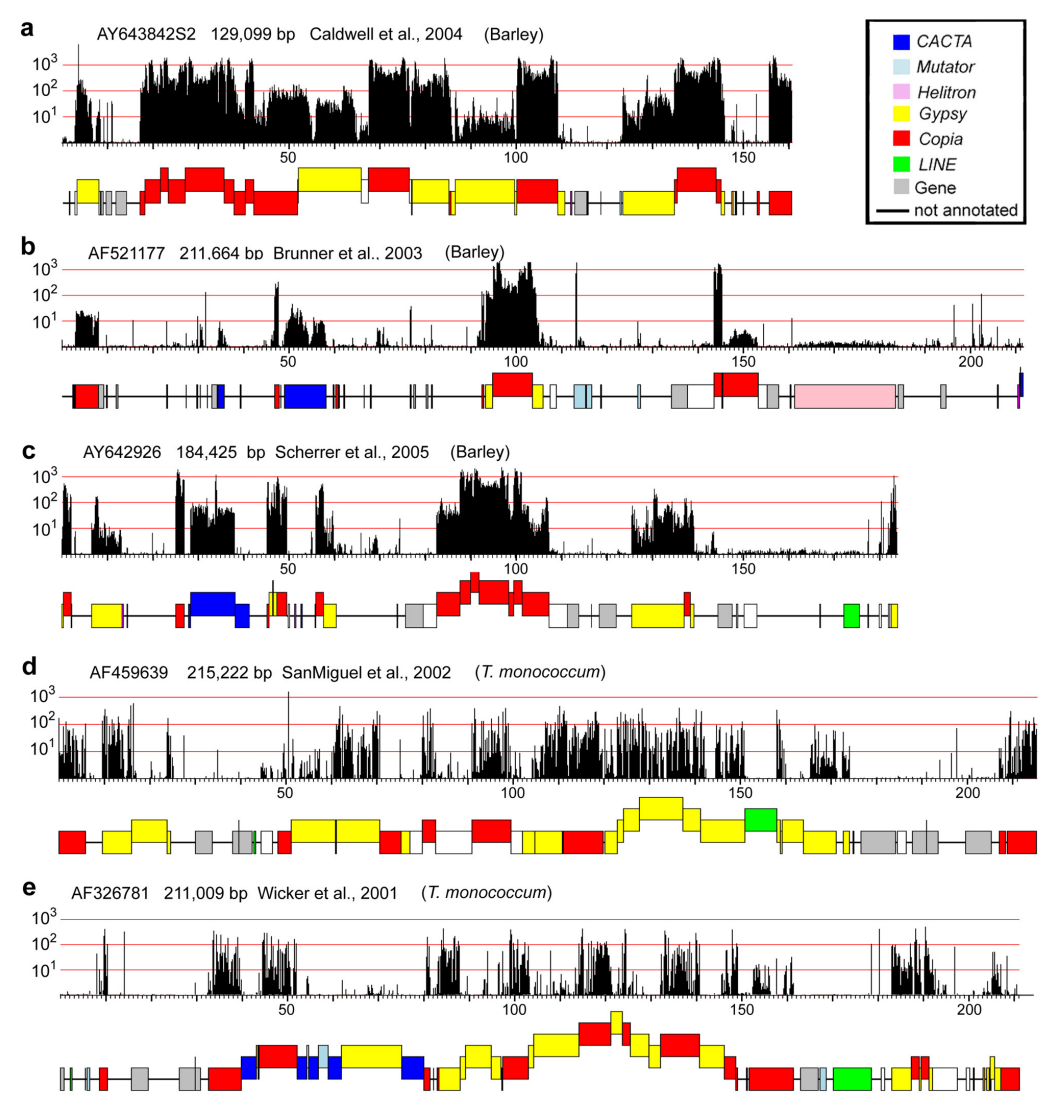
**MDR plots of publicly available sequences and their corresponding expert annotations.** The MDR plots at the top of each panel indicate the coverage with 20-mers at each position of the sequence. Note that the scale for the MDR signal is logarithmic. The corresponding expert annotation is displayed underneath the plot. TEs are indicated as coloured boxes with each colour corresponding to a TE superfamily. Nested TEs are raised above those into which they have inserted. The highly abundant elements in (a) at positions 17 kb – 38 kb (and all others with the same MDR signal strength) represent BARE1 elements, the most abundant TE in barley. a. through c. represent sequences from barley while d. and e. are sequences from einkorn wheat *Triticum monococcum*. Note that the MDR signal is much weaker in the *T. monococcum *sequences.

The standard sequences were subjected to MDR analysis to produce an index of repetitiveness for each 20-mer in a sequence. These data were then used to generate graphical representations ("MDR plots") which allow an easy and intuitive visual identification of repetitive regions on a sequence. For most MDR plots in this study, we chose a logarithmic scale to represent the repetitiveness of sequences (e.g. in Figure 2) because copy number among repetitive elements in large genomes can vary from a few copies to tens of thousands.

The expert annotation of each sequence was compared with its respective MDR plot, graphically and quantitatively. Three examples of graphical comparisons in barley are shown in Figure 2a through 2c. The complete dataset for the standard sequences is available as supplementary material (see Additional files 3 and 4). The graphical comparison shows that, over the majority of the data, the MDR plots confirm the results of expert annotations, as almost all regions that were annotated as TEs also showed clear signals in the MDR plot. The borders of individual TEs are easily recognisable as sharp changes in the intensity of MDR signals (Figure 2a–c). Some regions show MDR signals where no TEs were annotated: these represent novel repeated sequences (see below).

In a quantitative analysis, we calculated the percentage of all standard sequences that were classified as repetitive by both expert annotation and MDR analysis. As a threshold, we considered as repetitive all regions that showed an MDR coverage higher than 2 as being repetitive. The rationale for that was that our Illumina/Solexa dataset provided an approximately 10% coverage a genome equivalent. Thus, one out of 10 single copy sequences is expected to be covered. Sequences with 10 copies are, on average, covered one-fold and those with 100 copies 10-fold. The coverage with Solexa reads is mathematically equivalent to the MDR coverage. For this study, we considered an MDR signal of 2 as repetitive (corresponding to approximately 20 copies in the genome) and sequences that were covered less than 2-fold by MDR were considered low-copy.

An average of 70.2% of the standard sequences was identified as repetitive by expert annotation (Table 3). In contrast, MDR analysis identified consistently a smaller area with an average of 49.5% as representing repetitive DNA (ranging from 23.3 to 63.3, Table 3). This lower value can be explained by two factors. First, variable regions within TEs can have very low MDR coverage but are easily identified as parts of the respective TE by expert annotation (because they lie within the boundaries and do not extend the overall expected size of the respective TE). In the graphical representation, the borders of most TEs can still be clearly identified in the MDR plot, despite regions of lower coverage (see below). Thus, the overall visually apparent coverage with MDR signals coincides well with the expert annotation. Second, several of the standard sequences contain apparently low-copy TEs which could still be identified based on structural features such as Long terminal repeats (LTRs), Terminal inverted repeats (TIRs) or coding regions during expert annotation. For example, DNA transposons of the *Mutator *superfamily usually contain large (several hundred bp) TIRs and are flanked by a 9 bp target site duplication. Such elements can be easily recognised even if they show now sequence homology to known TE families (e.g. the non-autonomous Sukkula elements can be identified solely based on their canonical LTR retrotransposon structure, [25].

**Table 3:** Comparison of the fractions that were identified as repetitive by manual annotation and through MDR analysis.

Sequence	Exp^1^	MDR^2^	OL^3^	New^4^	Total^5^
AF474373	56.8	48.1	40.9	7.1	63.9
AF521177	37.4	23.3	18.5	4.8	42.2
AY268139	71.7	58.6	55.6	3.0	74.7
AY485643	44.4	38.2	30.8	7.4	51.8
AY642926	48.1	38.0	32.9	5.1	53.2
AY643842S2	71.7	50.5	48.6	1.9	73.6
AY643842S3	79.9	69.3	68.0	1.3	81.2
AY661558	87.6	62.1	59.6	2.5	90.1
EF067844	81.5	57.7	56.0	1.7	83.2

Average	64.3	49.5	45.6	3.9	68.2

All figures are in % of the total length of the sequence analysed.

^1^Repetitive fraction identified by expert annotation.

^2^Repetitive fraction identified by MDR analysis.

^3^Overlap, fraction covered by both.

^4^Fraction identified as repetitive exclusively through MDR.

^5^Expert annotation and MDR analysis combined

Interestingly, the results of expert annotation and MDR analysis do not completely overlap as all sequences contain a considerable fraction of 1.3% – 7.1% (average 3.9%) which was not recognised as repetitive in the expert annotation but shows clearly visible MDR signals. Most of these regions have moderate to low copy numbers and, thus, most likely contain novel types of TEs (see below).

### Barley MDR signal intensity decreases strongly if applied to analysis in other Triticeae species

To determine the usefulness of barley genomic shotgun sequence information in predicting repetitive DNA in other Triticeae species, we performed an analogous MDR analysis by appying the same Illumina/Solexa dataset on a number of publicly available sequences from a diploid wheat species (*Triticum monococcum *L., einkorn). These were expert annotated in detail to the same quality as the set of standard sequences from barley described above. Wheat and barley diverged from each other approximately 11 MYA [26]. The comparison of MDR plots with the expert annotation showed that both the density and the intensity of MDR signals on *T. monococcum *is much decreased compared to those obtained for the barley sequences. As shown in Figure 2d and 2e, MDR signals are distributed somewhat sporadically across the repetitive portions of the two *T. monococcum *sequences. High-copy repeats such as *Angela *and *WIS *(the wheat homologs of *BARE elements*) still produce relatively strong and consistent signals, however, roughly an order of magnitude weaker than in barley. Less abundant and diverse repeats such as *CACTA *transposons, *LINE*s or low-copy *Gypsy *elements produce signals that are barely (if at all) above the threshold for being recognised as repeats (Figure 2d and 2e). The complete set of MDR plots for wheat sequences is available as supplementary material (see Additional file 5).

### Identifying the putative gene space by combining automated repeat masking with MDR analysis

**Figure 3:**
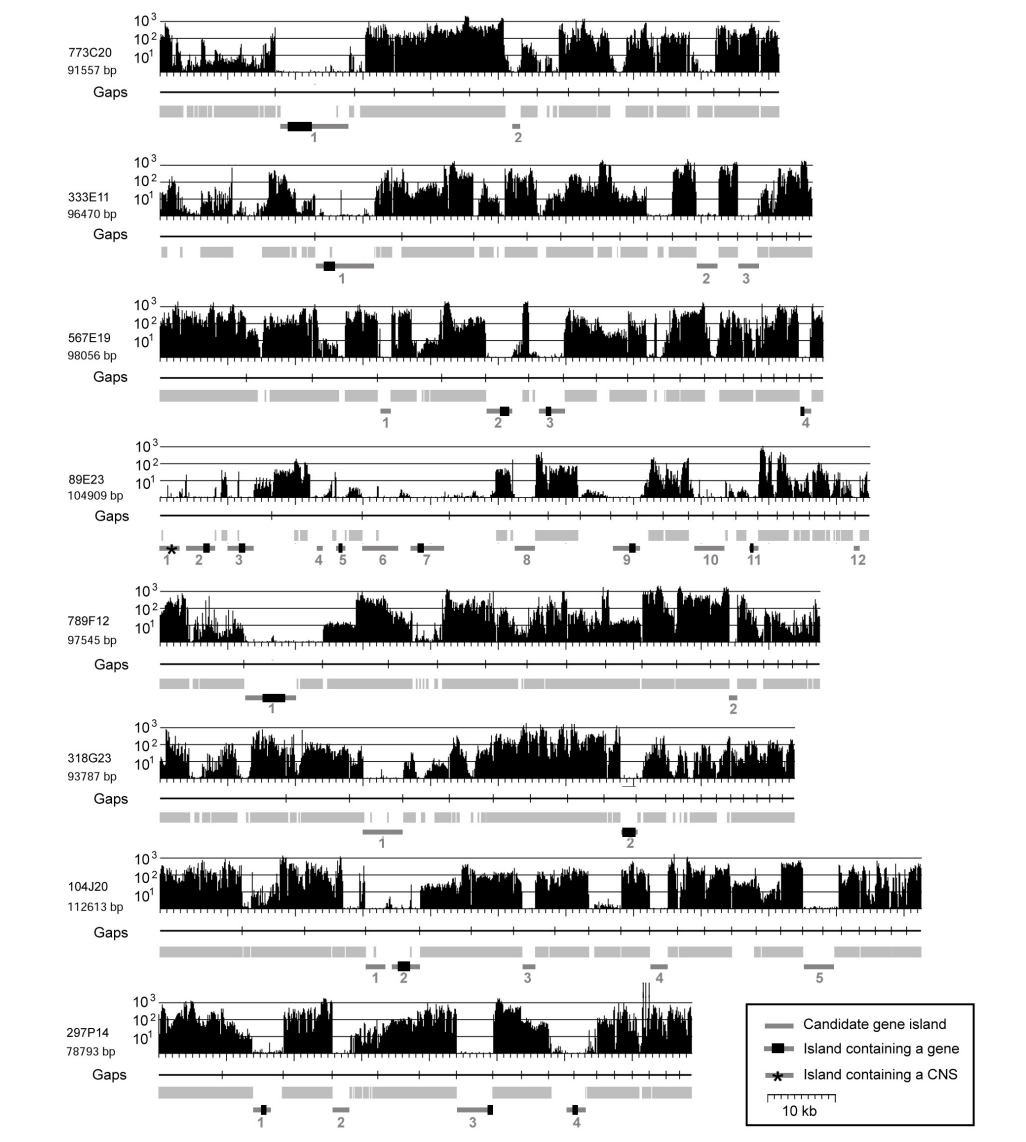
**Detection of gene-containing portions of low-pass survey sequenced BAC clones.** For all, the MDR plot is indicated at the top. Underneath, the positions of Gaps in the sequence are indicated as vertical bars in horizontal black lines. Regions that were repeat masked are indicated as light grey boxes. Candidate gene islands are indicated at the bottom as dark grey bars with genes indicated as black boxes (CNS: conserved non-coding sequence).

To test whether MDR analysis can assist gene identification and repeat masking in unfinished genomic sequences of barley, we *de novo *survey sequenced eight BAC clones to obtain a set of sequences whose composition is entirely unknown and thus would allow for an unbiased approach. The shotgun sequences of the eight BAC clones were used for an initial assembly resulting in 16 to 23 unordered contigs per BAC insert (Figure 3). The sequence contigs of each BAC were concatenated and separated by stretches of 100 N's and no efforts were made to arrange the sequence contigs in their proper physical linear order.

Repeat masking was done both at the DNA and protein level against the complete TREP nucleotide database and its protein section (PTREP), resulting in an average of 62.8% and 25.8% of the sequences being masked (Table 4). The regions identified as repetitive by MDR analysis and repeat masking approaches largely overlap. The combined data resulted in 66.1% masked sequences (Table 4). Again, MDR analysis identified a lower average fraction as repetitive (50.8%) than repeat masking, but a considerable fraction of 4.7% was unique to MDR analysis (Table 4). The combination of both repeat masking and MDR datasets identified an average of 70.7% of the BAC sequences as repetitive. Seven BACs contained between 65.9% and 81.7% repetitive sequences and one (89E23) contained only 38.1% (Table 4).

**Table 4:** Comparison of fractions of de novo sequenced BACs that were identified as repetitive by repeat masking and MDR analysis.

BAC	size	BLASTN^1^	BLASTX^2^	combined^3^	MDR^4^	combined^5^	New^6^
773C20	91561	67.5	32.6	71.4	52.3	74.4	3
333E11	96473	57.0	23.0	59.5	47.8	65.9	6.5
567E19	98005	69.3	25.8	71.2	60.1	76.5	5.3
89E23	104909	26.8	9.7	30.1	25.5	38.1	8
789F12	97547	73.9	36.7	79.7	57.2	81.7	2.1
318G23	93787	67.3	20.1	70.6	53.1	77.1	6.5
104J20	112610	72.9	33.8	77.4	55.5	80.5	3.2
297P14	78796	70.9	24.6	71.0	57.6	73.6	2.6

Total	773688	62.8	25.8	66.1	50.8	70.7	4.7

All figures are in % of the total length of the sequence analysed.

^1^Fraction masked based on BLASTN search against TREP

^2^Fraction masked based on BLASTX search against PTREP

^3^Fraction masked when information form BLASTN and BLSTX is combined

^4^Repetitive fraction identified by MDR analysis

^5^Fraction masked when information form BLAST and MDR is combined

^6^Fraction identified as repetitive exclusively through MDR

As immediate candidates for gene-containing regions, we considered all regions that were at least 1 kb in size, were not repeat masked and showed no or very low MDR signals (Figure 3). Small sharp peaks of strong MDR signals in candidate gene islands were ignored as they likely represented miniature inverted-repeat transposable elements (MITEs) or microsatellites, frequently found inside or nearby genes in Triticeae [27]. In total, 34 such candidate regions were identified in the eight BACs. Seven BACs contained between two and five, while 89E23 contained 12 – consistent with its low repeat content (Figure 3).

In 17 of the candidate gene islands, putative coding sequences of genes were found. In two cases (BAC 567E19, island 2 and 4 and BAC 297P14 island 3 and 4), a single gene was split and found on two different sequence contigs which was due to the low-pass sequence status of the BAC clones. One candidate gene island contained no protein coding sequences but a conserved non-coding sequence (CNS, BAC 89E23, island 1, Figure 3). We characterised it as CNS because it contains no homology to protein coding sequences, and no open reading frame could be detected by means of gene prediction algorithms and yet it is highly conserved at the DNA level to rice genomic sequences. The CNS has a size of 207 bp and is 92% identical between rice and barley. In the rice genome, we found nine copies of that sequence. Interestingly, the CNS appears to be expressed as we found a barley EST which is 97% identical to it.

The large overlap in repetitive sequences identified by MDR or "traditional" repeat masking implies that in case of a lack of a comprehensive reference repeat database, as it is available for barley, a simple MDR analysis of a new non-annotated sequence would be sufficient to identify all potential genic regions and no additional repeat masking would be necessary.

### MDR plots of individual TEs reveal variable regions and over- and under-represented motifs

**Figure 4:**
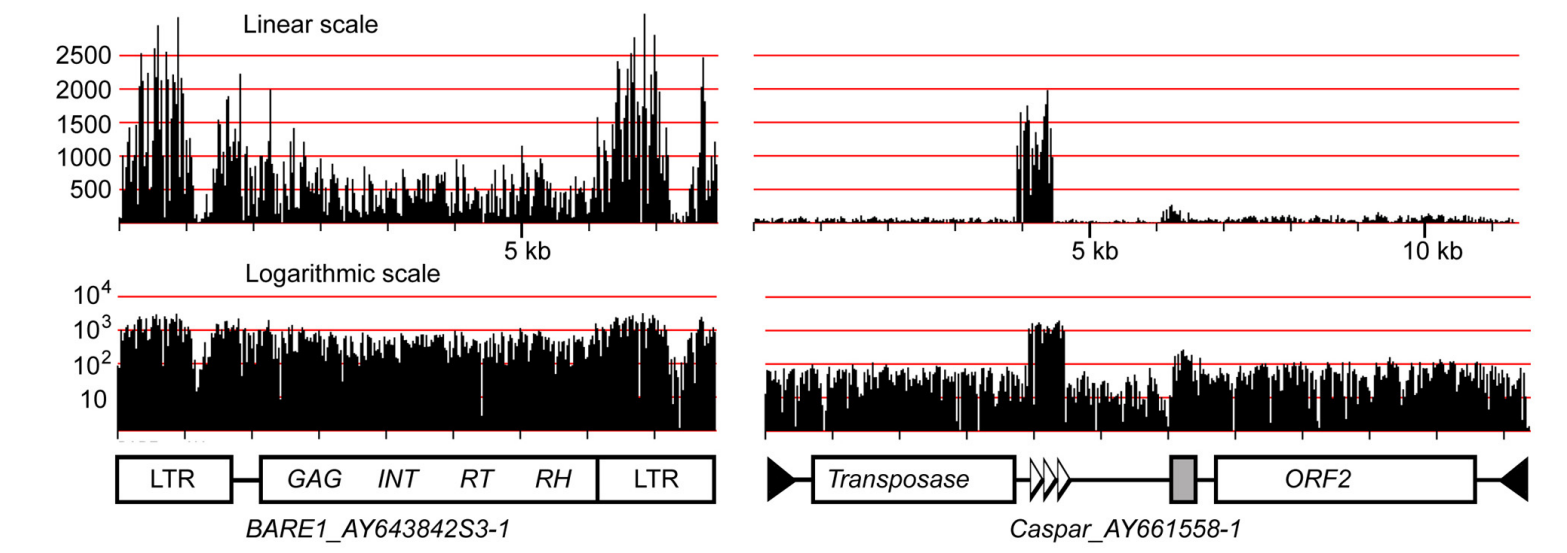
**MDR plots of a *BARE1 *LTR retrotransposon and a *Caspar *DNA transposon.** The linear scale (top) illustrates the strong variation in relative abundance between the two elements but also of different regions within the two elements. The LTRs of *BARE1 *are roughly 3-fold over-represented whereas a region containing tandem repeats in the *Caspar *element are at least 20 times more abundant than the rest of the element. The grey box indicates a region of low-complexity DNA. The logarithmic representation allows an easy identification of variable regions (e.g. in the *BARE1 *LTR and between the two CDS in *Caspar*).

To study their characteristics and abundance, we extracted all individual annotated TEs from BAC sequences and linked them to their respective MDR profile. Figure 4 shows examples of *BARE1 *and *Caspar *elements. The MDR plot of *BARE1_AY643842S3-1 *showed that the two LTRs are 3–4 times more abundant at genome-scale than the internal domain of the element. This over-representation of LTR sequences can be explained with the simple fact that a full-length *BARE1 *element contains two LTRs and that the barley genome contains many solo-LTRs, resulting from intra- or inter-element non-homologous recombination [3,28].

Similarly, *Caspar *(and other *CACTA*) transposons contain regions with much higher copy numbers than the overall average for the whole element (Figure 4b), because of the presence of large arrays of tandem repeats. Previous studies showed that *CACTA *transposons can contain arrays with dozens of such repeat units [29], some of which were initially described as *Afa *repeats [30,31]. Thus, the number of individual repeat units can by far exceed the actual copy number of the respective transposon. Additionally, *Caspar *transposons usually contain at least one region of several hundred bp which consists almost exclusively of low-complexity G/A-rich motifs. This region can also be easily identified in the MDR plot in Figure 3a.

MDR plots of individual TEs can also be used to identify variable regions. For example, the LTR of *BARE1 *showed a low coverage between positions 1000–1400. This indicates a variable region in the otherwise highly conserved LTR sequence. Indeed, comparison of 19 *BARE1 *LTR deposited at TREP showed much higher sequence variability in that region (data not shown). The *Caspar *transposon too has a region with lower sequence conservation between the two coding regions. This is also confirmed by comparison of different copies of *Caspar *elements (data not shown).

### Identification of novel repeats based on MDR plots

Visual inspection of the graphical output of the standard set and the newly sequenced BACs yielded several regions that showed a clear MDR signal but for which no TE sequences were annotated. The first was located close to the right end of sequence AF521177 and contained the only *Helitron *found so far in barley (Figure 2b). This element was originally identified as an insertion in orthologous loci between two barley varieties Morex and Cebada Capa [32]. However, it was previously unclear if (i) the entire 22 kb insertion indeed consisted of a single element or whether it (ii) contained nested insertions of other, yet unknown, TEs or (iii) fragments of non-TE genes, which *Helitrons *often carry [33]. In the present analysis, the MDR plot showed for the entire element a relatively even signal corresponding to a 2–3-fold coverage with Illumina/Solexa reads (linear scale), indicating that it is present in this form in about 20–30 copies in the barley genome.

Similarly, a region of more than 20 kb close to the right end of the Cebada Capa sequence AY642926 (approx positions 150 kb – 170 kb) showed an even MDR signal, indicating the presence of a very large TE with moderate copy number. Indeed, comparison of that region with its ortholog in Morex led to the identification of an exotic TE with a size of more than 28 kb. It contains a transposase that resembles slightly those of *Mutator *elements but it does not contain any of the typical structural features such as terminal inverted repeats or target site duplication. Thus, its classification remains uncertain yet.

A third interesting repeat was found in an intron of a gene of the *de novo *survey sequenced BAC 798F12 (gene island 1, Figure 3). Specific analysis of the region revealed it to be a short insertion of a fragment of mitochondrial DNA. Insertions of fragments of organellar DNA into the nuclear genome of plants are a frequently observed phenomenon [34]. The inserted fragment is part of the mitochondrial MatR gene which contains a reverse transcriptase domain and is, according to gene ontology, involved in RNA splicing and RNA-dependent DNA replication.

In order to systematically extract all novel repeat sequences from the standard set, a Perl program was written to recognize regions with strong MDR signals which were not identified by expert annotation. We were able to identify by this approach 62 novel repeats which ranged in size from 82 to 1999 bp (see Additional file 6). Three of them simply represented microsatellite sequences and were excluded. The average MDR coverage of the other 59 repeats (i.e. the average strength of the MDR signal across the whole element) ranged from 1.7 up to 142, indicating that some are low-copy and other are rather high-copy repeats. The low coverage of less than 2 for some repeats is due to the fact that, although we required a minimum MDR coverage of 2 for a sequence to be considered repetitive, we still allowed stretches with lower coverage within the repeat (see methods). All 59 novel repeats were used in BLASTN searches against a publicly available set of 315 genomic Triticeae sequences larger than 20 kb. Most of the repeats were present in multiple copies, thus high MDR coverage mostly correlated with a high copy number in the public dataset.

## Discussion

The focus of this study was to evaluate the potential of short read sequencing (SRS) in combination with mathematically defined repeat (MDR) analysis for surveying and draft annotating repetitive DNA in the large and complex barley genome. Differing from previous studies which have explored the potential of genomic 454 sequencing for stretches of plant genomic DNA packed with repetitive elements of different kind [35] and in purpose of characterising the global repetitive DNA landscape of legume genomes [36,37], we studied the usefulness of Illumina/Solexa sequencing which produces a higher number of bases per run but much shorter reads than 454. Barley and other Triticeae species can serve as an appropriate training system for such an evaluation because comprehensive knowledge of the TE composition of their genomes is available and has cumulated to formation of a high-quality Triticeae repeat database [6]. Thus, the pre-existing information of accurately annotated full length repeat elements allowed for the detailed and comprehensive comparison with the generated MDR data. The observed strong regional overlap between the statistical survey for MDR distribution with repeats identified by expert annotation, imply that even for organisms without significant genomic sequence information available, a limited investment into SRS genomic shotgun sequencing allows to accumulate a large amount of valuable biological information which can be efficiently exploited for comprehensive repeat and putative gene space identification.

MDR plots also allow to specifically focus on repeat sequences that are likely of interest to a particular research project. For example, it could be important to know whether a gene family contains TEs in promoter, intron or upstream/downstream regulatory sequences as they could affect expression of the gene. MDR analysis would thus allow to quickly identify most repetitive elements in and around genes of interest.

The comparison between MDR analysis and expert annotation in barley clearly demonstrated that manual annotation, if carefully done, can identify almost all TEs present in a sequence. However, this requires a critical amount of available training data for the organism of interest. Our experiences with the highly repetitive genomes of wheat and barley show that several hundred kb of large genomic sequences are required to identify most major high-copy TE families. Indeed, the main repeat types of wheat and barley were all discovered early on in only a few large genomic sequences [28,38-40]. Thus, as an important outcome of the present study we can conclude that the accumulated knowledge of barley repetitive elements has reached already a very comprehensive level in the available repeat databases. The generated MDR data helped demonstrating that expert annotation of TEs can reach a high level of completeness, as the repeat identification by MDR analysis revealed in barley only relatively few new repeat types. This information is particularly valuable for sequences like AF521177 (Figure 2b) which has a very low repeat content. Without having access to statistical repeat prediction by MDR analysis we previously assumed that the sequence contained a substantial proportion of yet unknown TE sequences. The comparison to MDR plots now provided confirmation that AF521177 in fact bears extensive regions which neither contain genes nor TEs. The obvious bottleneck of manual annotation is that it is labour-intensive and time-consuming and requires a lot of expert knowledge.

### Automated repeat masking and MDR analysis both provide rapid and reliable ways to determining the low-copy fraction and putative gene space in highly repetitive BAC clones

We assumed that for most projects, researchers will be interested mainly in the gene content of a particular BAC instead of fine annotation of the full repeat composition and organization. Thus, an initial search for gene containing regions will be the first step of most analyses, before efforts would be undertaken towards finishing the BAC sequence and its annotation. Our approach of combining simple repeat masking with MDR data proved to be an efficient way of identifying gene containing sequences. Exactly half of the candidate gene islands that were identified this way indeed contained (non-TE) coding sequences. If unmasked genomic sequences were used for gene identification, for example, in a BLAST search against rice proteins, interpretation of outputs tend to be labour-intensive as most of the coding sequences that produce BLAST hits will belong to TEs and the few actual genes might be missed.

Our data showed there is a great overlap in repetitive regions identified by MDR and "traditional" repeat masking. Thus, for the reliable identification of potential gene islands both methods perform similarly well. The advantage of MDR analysis is that an MDR index can be constructed from a Illumina/Solexa dataset in a relatively short time at limited investment without going through the labour-intensive process of constructing a repeat library for the species of interest.

An important result of the repeat masking *vs*. MDR analysis experiment was the identification of a large conserved non-coding sequence (CNS) that occupied one of the candidate gene islands. CNS were described previously in mammals as gene-regulatory sequences [41] or as remnants of sequence identity due to lack of divergence time [42]. In plants, CNS function is still unclear [43,44] and the average motif length was usually found to be much smaller [43]. The approach described here provides the means to identify such sequences at a large scale and in large numbers.

Although sample size of genomic sequences considered for this study is small, our results suggest that the barley genome contains relatively low amounts of low-copy DNA which does not encode proteins. Nevertheless, one still has to expect many regions with no obvious coding capacity as half of the candidate gene islands on the *de novo *sequenced BACs harboured no genes. Another example is the sequence AF521177 which contains large regions where neither genes nor TEs were found. Such low-copy islands may still contain TE sequences that have fewer than 20 copies in the genome and are thus not detected at the 0.1 X coverage provided by the Illumina/Solexa data of our study. Lower abundance repeats could probably be identified by increasing the genome coverage with Illumina/Solexa reads. However, at such low-copy numbers, the borderline between genic sequences (e.g. multi-copy gene families) and TE and other selfish sequences may be blurred.

Some of the low-copy sequences might also represent highly degenerate TE sequences which have lost most sequences homology due to a long time of degeneration. We expect that fraction to be minimal, because intergenic sequences in Triticeae are rapidly turned over through permanent creation of DNA by TE amplification and DNA loss through illegitimate recombination and unequal crossing-over [3,28,38,45]. Thus, hardly any TEs older than 3–4 Million years are found [38,45,46].

### Characterisation of novel repeats

The availability of a highly curated set of barley sequences allowed the automated isolation of novel repeat sequences by simply using strong MDR signals outside of annotated TEs as indicator of repetitiveness. This led to the rapid identification of 60 novel TE sequences. In addition to the typical and highly abundant plant TEs (e.g. LTR retrotransposons), the barley (or any plant) genome contains a multitude of TE-derived sequences that do not contain typical and easily identifiable structural features or coding regions [25,29]. Many of them have only a moderate copy number per genome and are therefore not detectable if only a limited dataset, i.e. publicly available large genomic Triticeae sequences, is being used, or if closely related orthologous regions are analyzed and compared [47,48]. MDR data derived from whole genome SRS allows for the identification of novel elements merely on the basis of copy number. Since the described approach is a quantitative assessment and no qualitative interpretation, the identified novel repeats have to be analysed further and characterised in more detail in order to sustain the discovered TE information for future sequence annotations.

Already simple visual inspection of MDR plots and comparison to expert annotation or repeat masking results allows identifying possible novel repeat regions. Such an approach led to the characterisation of a novel large 28 kb TE, which was identified solely due to the information from MDR analysis. As this element was not found in any other publicly available sequence, MDR data was the only indication for it being indeed a multi-copy sequence. Additionally, a small sharp spike in MDR signal within an intron led to the rapid identification of a small mitochondrial insertion. Overall, MDR analysis in barley provided relatively few novel repeat sequences, but this is mainly attributable to the already well documented repeat landscape of the Triticeae. Thus, if the same approach would be undertaken in a species with a less well characterized repetitive fraction, a single Illumina/Solexa run covering 10% of the genome could help identifying most of the main repeat types when matched against a large genomic (e.g. BAC) sequence. Probably, the novel repetitive elements identified in this study could have been discovered as well through exhaustive TE annotation and comparison with other publicly available sequences. However, the obvious advantage of MDR analysis over manual annotation is its much lower time requirement.

## Conclusion

Low-pass short-read-genomic shotgun sequencing of approximately 10% of a haploid genome equivalent allowed rapid and easy identification of the low-copy fraction (i.e. putative gene space) and repetitive DNA regions in barley. Converted into an index of mathematically defined repeats (MDR) the low-pass genomic shotgun sequence dataset proved to be as efficient as traditional repeat masking based on BLAST searches. In addition, it proved very efficient in the identification of novel repeat types thus further refining our knowledge on the organization of the barley genome. The obvious advantage of MDR over the traditional methods of repeat masking and expert annotation is its lower time and pre-existing expert knowledge requirement, as no repeat databases have to be constructed and *de novo *repeat annotation (which is inherently time-consuming and labour-intensive) becomes fast and easy. An MDR index produced from SRS reads thus provides an elegant tool for the identification of repetitive and low-copy regions in genomic sequences. An experiment as described in this study takes about one to two weeks and can generate the necessary dataset for comprehensive repeat prediction by MDR for any chosen genome. As sequencing becomes cheaper and more and more efficient, similar low-effort datasets for genome analysis and comparative genomics could be obtained for a multitude of species for which no or only few sequence data is or will become available in short time.

## Authors' contributions

TW provided expert annotation of barley BACs, performed MDR/expert annotation comparisons and drafted the manuscript. AN and JS contributed to the development of the MDR matrices and the analysis of the BACs. FS provided expert annotation of the wheat BACs. VG identified and selected BAC clones for draft sequencing. DW contributed to the experimental design. NS conceived the study and contributed to writing of the manuscript. All authors contributed to the reading and approval of the manuscript.

## Supplementary Material

Additional file 1 **Supplementary Table 1.** List of accession numbers of 315 publicly available large genomic sequences from Triticeae. Click here for file

Additional file 2 **Supplementary Figure 1**. MDR plot of the barley chloroplast sequence. The sequence is covered with Solexa reads approximately 60-fold. A region that is present in two copies in reverse orientation is clearly identifiable through its higher coverage. The inversion is indicated with arrows underneath the map. Click here for file

Additional file 3 **Supplementary Figure 2**. MDR plots of publicly available sequences from barley and their corresponding expert annotations. The MDR plots at the top indicate the coverage with 20-mers at each position of the sequence. Note that the scale for the MDR signal is logarithmic. The corresponding manual annotation is displayed underneath the plot. TEs are indicated as coloured boxes with colours corresponding to superfamilies. Nested TEs are raised above the ones into which they have inserted. Click here for file

Additional file 4 **Supplementary Figure 3**. MDR plots of publicly available sequences from barley and their corresponding expert annotations. The MDR plots at the top indicate the coverage with 20-mers at each position of the sequence. Note that the scale for the MDR signal is logarithmic. The corresponding manual annotation is displayed underneath the plot. TEs are indicated as coloured boxes with colours corresponding to superfamilies. Nested TEs are raised above the ones into which they have inserted. Click here for file

Additional file 5 **Supplementary Figure 4**. MDR plots of publicly available sequences from *Triticum monococcum *and their corresponding expert annotations. The MDR plots at the top indicate the coverage with 20-mers at each position of the sequence. Note that the scale for the MDR signal is logarithmic. The corresponding manual annotation is displayed underneath the plot. TEs are indicated as coloured boxes with colours corresponding to superfamilies. Nested TEs are raised above the ones into which they have inserted. Click here for file

Additional file 6 **Supplementary Table 2**. Novel repeats identified in the standard set of barley sequences. Click here for file
