# Supplementary material for: Low-pass shotgun sequencing of the barley genome facilitates rapid identification of genes, conserved non-coding sequences and novel repeats
Source: BMC Genomics. 2008 Oct 31;9:518. doi: 10.1186/1471-2164-9-518 (PMC2584661; doi:10.1186/1471-2164-9-518)
Supplement: Additional file 1 — Supplementary Table 1. List of accession numbers of 315 publicly available large genomic sequences from Triticeae. [file 1471-2164-9-518-S1.doc]

**Supplementary Table 1.** List of accession numbers of 315 publicly available large genomic sequences from Triticeae

EF426565

EF426564

EF567062

AC207962

AC207961

AC207960

AC207959

AC207958

AC207957

AC207956

AC207955

AC207954

AC207953

AC207952

AC207951

AC207950

AC207949

AC207948

AC207947

AC207946

AC207945

AC207944

AC207943

AC207942

AC207941

AC207940

AC207939

AC207938

AC207937

AC207936

AC207935

AC207934

AC207933

AC207932

AC207931

AC207930

AC207929

AC207928

AC207927

AC207926

AC207925

AC207924

AC207923

AC207922

AC207921

AC207920

AC207919

AC207918

AC207917

AC207916

AC207915

AC207914

AC207913

AC207912

AC207911

AC207910

AC207909

AC207908

AC207907

AC207906

AC207905

AC207904

AC207903

AC207902

AC207901

AY146587

AC200851

AC200850

AC200849

AC200848

AC200847

AC200846

AC200845

AC200844

AC200843

AC200842

AC200841

AC200840

AC200839

AC200838

AC200837

AC200836

AC200835

AC200834

AC200833

AC200832

AC200831

AC200830

AC200829

AC200828

AC200827

AC200826

AC200825

AC200824

AC200823

AC200822

AC200821

AC200820

AC200819

AC200818

AC200817

AC200816

AC200815

AC200814

AC200813

AC200812

AC200811

AC200810

AC200809

AC200808

AC200807

AC200806

AC200805

AC200804

AC200803

AC200802

AC200801

AC200800

AC200799

AC200798

AC200797

AC200796

AC200795

AC200794

AC200793

AC200792

AC200791

AC200790

AC200789

AC200788

AC200787

AC200786

AC200785

AC200784

AC200783

AC200782

AC200781

AC200780

AC200779

AC200778

AC200776

AC200775

AC200774

AC200773

AC200772

AC200771

AC200770

AC200769

AC200768

AC200767

AC200766

AC200765

EF560592

EF540321

AM050695

AM050694

AM050693

AM050692

AM050691

AM050690

AM050689

AM050688

AM050687

AM050686

AM050685

AM050684

AM050683

AM050682

AM050681

AM050680

AM050679

AM050678

AM050677

AM050676

AM050675

AM050674

AM050673

AM050672

AM050671

AM050670

AM050669

AM050668

AM050667

AM087558

AM087557

AM087556

AM087555

AM072971

AC200777

NC_002762

EF081031

EF081030

EF081029

EF081027

EF081026

EF081025

EF067844

AF325196

DQ900687

DQ900686

DQ900685

DQ899784

NC_008590

DQ871219

EF115541

DQ537337

DQ537336

DQ537335

CR626934

CR626933

CR626929

CR626926

Y14573

DQ995513

DQ469713

DQ469712

DQ351213

DQ767630

DQ767629

DQ767628

DQ767627

DQ767626

DQ767625

DQ767624

DQ767623

DQ767622

DQ767621

DQ767620

DQ767619

DQ767618

DQ767617

DQ767616

DQ767615

DQ767614

DQ767613

DQ767612

DQ767611

DQ767610

DQ767609

CT009735

CT009625

CT009588

CT009587

CT009586

CT009585

NC_007579

AP008982

DQ249273

AB042240

AY951945

AY951944

DQ267106

DQ267105

DQ267103

AY943294

AB027572

AY772735

AY772734

AY772733

AY772732

AY494981

AF474373

AY663392

AY485644

AY485643

AH013688

AY534123

AY534122

AY368673

AF488415

AF521177

AY268139

AY146588

AY188332

AF474072

AF326781

DQ157841

DQ157840

DQ157839

DQ157838

DQ157837

DQ157836

DQ157835

X13927

AY661558

AY641412

AY663391

AY642926

AY914086

AY914085

AY914084

AY914083

AY914082

AY914081

AY914080

AY914079

AY643844

AY643843

AH014393

AF497474

AF525764

AY491681

AF532104

AH012974

AY188333

AY188331

AF474071

AF474982

AF459639

AF446141

AF427791

AY013246

AF325198

AF325197

AF254799
