# Supplementary figures and images for: Low-pass shotgun sequencing of the barley genome facilitates rapid identification of genes, conserved non-coding sequences and novel repeats
Source: BMC Genomics. 2008 Oct 31;9:518. doi: 10.1186/1471-2164-9-518 (PMC2584661; doi:10.1186/1471-2164-9-518)

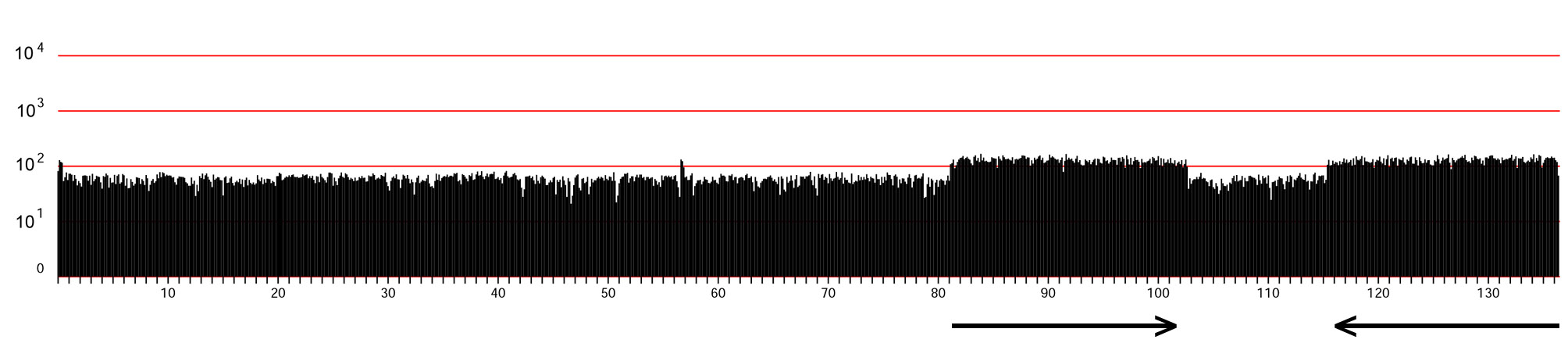

Supplement: Additional file 2 — Supplementary Figure 1. MDR plot of the barley chloroplast sequence. The sequence is covered with Solexa reads approximately 60-fold. A region that is present in two copies in reverse orientation is clearly identifiable through its higher coverage. The inversion is indicated with arrows underneath the map. [file 1471-2164-9-518-S2.doc]

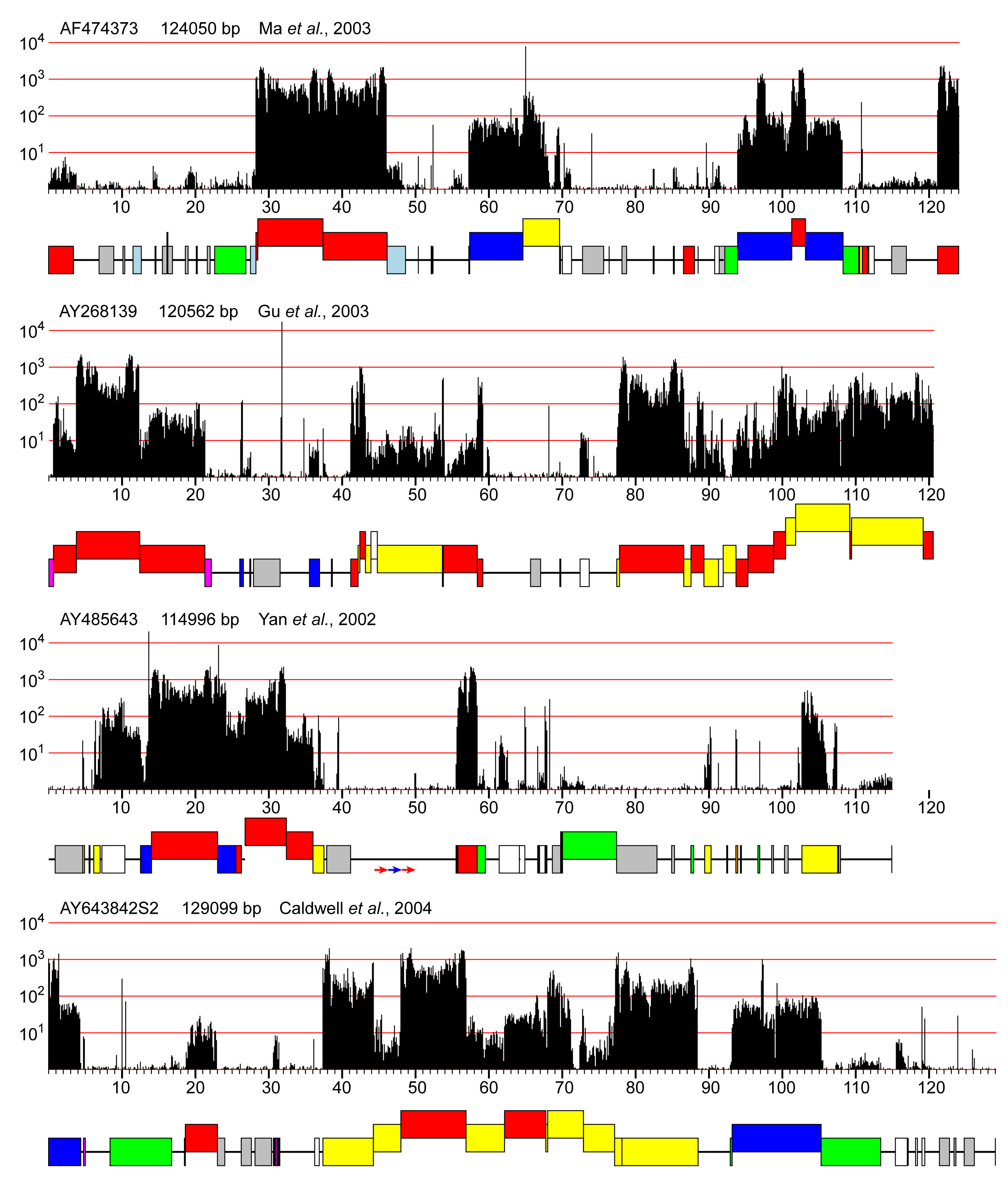

Supplement: Additional file 3 — Supplementary Figure 2. MDR plots of publicly available sequences from barley and their corresponding expert annotations. The MDR plots at the top indicate the coverage with 20-mers at each position of the sequence. Note that the scale for the MDR signal is logarithmic. The corresponding manual annotation is displayed underneath the plot. TEs are indicated as coloured boxes with colours corresponding to superfamilies. Nested TEs are raised above the ones into which they have inserted. [file 1471-2164-9-518-S3.doc]

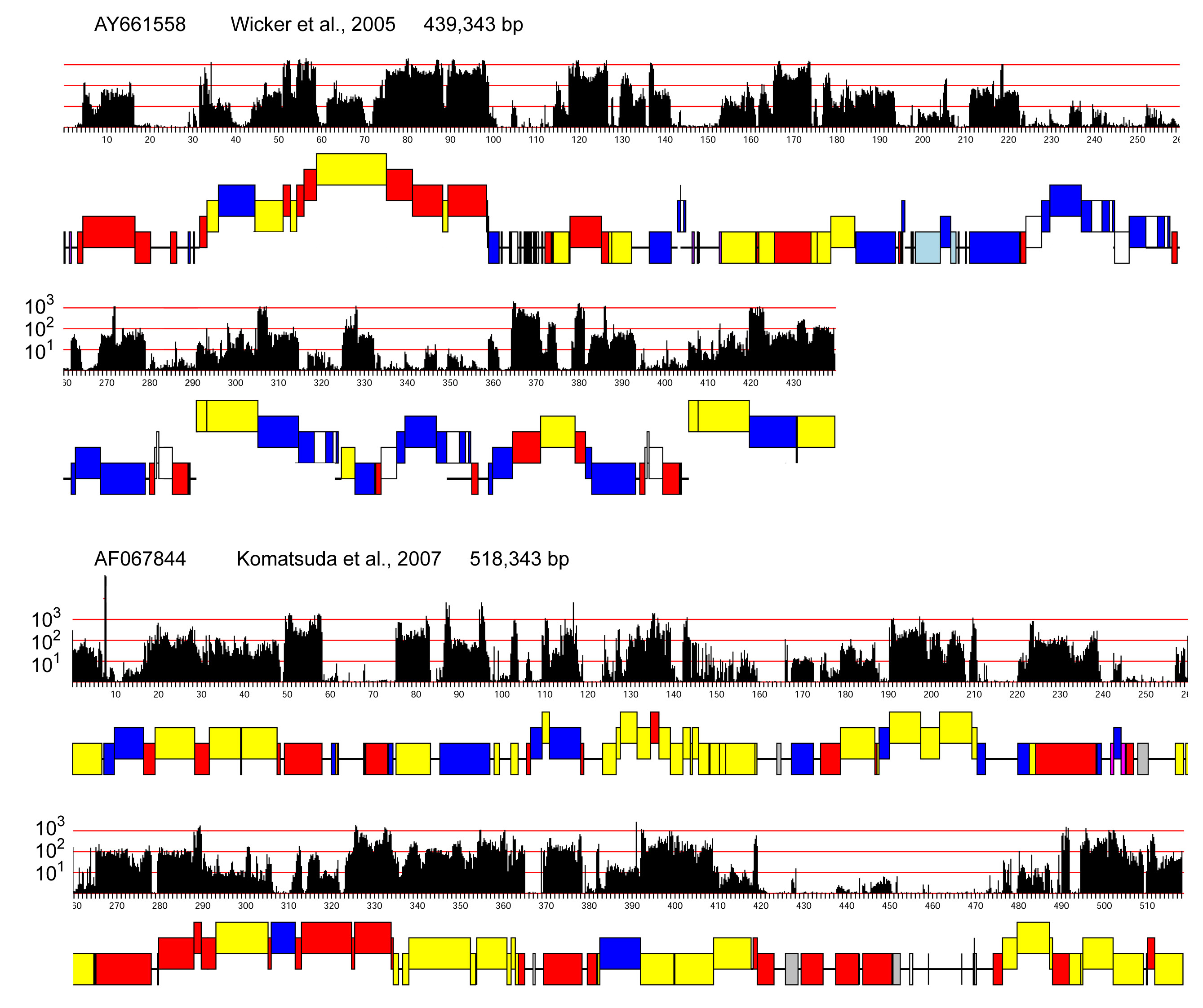

Supplement: Additional file 4 — Supplementary Figure 3. MDR plots of publicly available sequences from barley and their corresponding expert annotations. The MDR plots at the top indicate the coverage with 20-mers at each position of the sequence. Note that the scale for the MDR signal is logarithmic. The corresponding manual annotation is displayed underneath the plot. TEs are indicated as coloured boxes with colours corresponding to superfamilies. Nested TEs are raised above the ones into which they have inserted. [file 1471-2164-9-518-S4.doc]

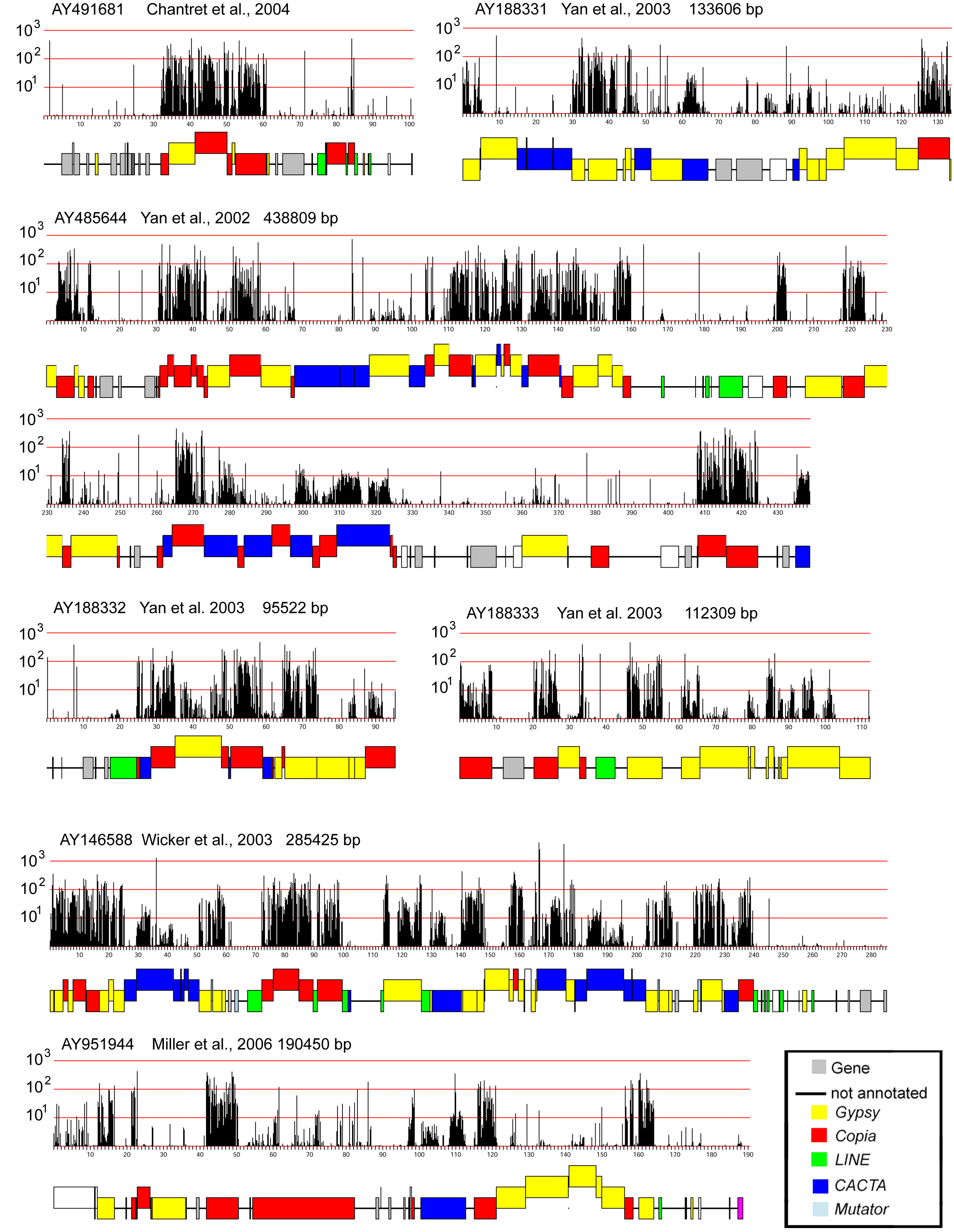

Supplement: Additional file 5 — Supplementary Figure 4. MDR plots of publicly available sequences from Triticum monococcum and their corresponding expert annotations. The MDR plots at the top indicate the coverage with 20-mers at each position of the sequence. Note that the scale for the MDR signal is logarithmic. The corresponding manual annotation is displayed underneath the plot. TEs are indicated as coloured boxes with colours corresponding to superfamilies. Nested TEs are raised above the ones into which they have inserted. [file 1471-2164-9-518-S5.doc]
