# Supplementary material for: Low-pass shotgun sequencing of the barley genome facilitates rapid identification of genes, conserved non-coding sequences and novel repeats
Source: BMC Genomics. 2008 Oct 31;9:518. doi: 10.1186/1471-2164-9-518 (PMC2584661; doi:10.1186/1471-2164-9-518)
Supplement: Additional file 6 — Supplementary Table 2. Novel repeats identified in the standard set of barley sequences. [file 1471-2164-9-518-S6.doc]

| **Supplementary Table 2.** Novel repeats identified in the standard set of barley sequences. | | | | | | |  |
| --- | --- | --- | --- | --- | --- | --- | --- |
| source | position | end | name | size | coverage | standard set | rem |
| AF474373 | 3514 | 3630 | AF474373_rep-1 | 116 | 2.1 | 4 |  |
| AF474373 | 14720 | 14841 | AF474373_rep-2 | 121 | 2.2 | 9 |  |
| AF474373 | 19158 | 19240 | AF474373_rep-3 | 82 | 3.0 | 1 |  |
| AF474373 | 19576 | 19711 | AF474373_rep-4 | 135 | 2.3 | 2 |  |
| AF521177 | 30669 | 30915 | AF521177_rep-1 | 246 | 5.5 | 6 |  |
| AF521177 | 30948 | 31134 | AF521177_rep-2 | 186 | 2.2 | 1 |  |
| AF521177 | 36012 | 36127 | AF521177_rep-3 | 115 | 2.3 | 0 | SSR |
| AF521177 | 46825 | 46908 | AF521177_rep-4 | 83 | 142.6 | 165 |  |
| AF521177 | 69397 | 69500 | AF521177_rep-5 | 103 | 6.7 | 2 |  |
| AF521177 | 69719 | 69875 | AF521177_rep-6 | 156 | 4.3 | 1 |  |
| AF521177 | 69911 | 70115 | AF521177_rep-7 | 204 | 3.7 | 2 |  |
| AF521177 | 70621 | 70743 | AF521177_rep-8 | 122 | 3.2 | 4 |  |
| AF521177 | 70816 | 70926 | AF521177_rep-9 | 110 | 8.1 | 4 |  |
| AF521177 | 91823 | 91925 | AF521177_rep-10 | 102 | 3.8 | 12 |  |
| AF521177 | 92108 | 92279 | AF521177_rep-11 | 171 | 4.4 | 27 |  |
| AF521177 | 92427 | 92518 | AF521177_rep-12 | 91 | 282.2 | 0 | SSR |
| AF521177 | 200773 | 200913 | AF521177_rep-13 | 140 | 2.3 | 22 |  |
| AF521177 | 202513 | 202708 | AF521177_rep-14 | 195 | 114.9 | 25 |  |
| AY485643 | 7033 | 7120 | AY485643_rep-1 | 87 | 24.9 | 16 |  |
| AY485643 | 10429 | 12428 | AY485643_rep-2 | 1999 | 39.1 | 24 |  |
| AY485643 | 12462 | 12587 | AY485643_rep-3 | 125 | 24.6 | 6 |  |
| AY485643 | 26360 | 26699 | AY485643_rep-4 | 339 | 73.8 | 7 |  |
| AY485643 | 39360 | 39587 | AY485643_rep-5 | 227 | 71.7 | 21 |  |
| AY485643 | 102126 | 102268 | AY485643_rep-6 | 142 | 14.2 | 5 |  |
| AY485643 | 114001 | 114105 | AY485643_rep-7 | 104 | 2.0 | 1 |  |
| AY642926 | 2859 | 3124 | AY642926_rep-1 | 265 | 6.1 | 6 |  |
| AY642926 | 55052 | 55317 | AY642926_rep-2 | 265 | 6.1 | 6 |  |
| AY642926 | 60925 | 61027 | AY642926_rep-3 | 102 | 2.2 | 2 |  |
| AY642926 | 62426 | 62526 | AY642926_rep-4 | 100 | 1.9 | 1 |  |
| AY642926 | 67906 | 68028 | AY642926_rep-5 | 122 | 4.7 | 3 |  |
| AY642926 | 68648 | 68731 | AY642926_rep-6 | 83 | 2.6 | 1 |  |
| AY642926 | 68894 | 69067 | AY642926_rep-7 | 173 | 3.2 | 1 |  |
| AY642926 | 69117 | 69226 | AY642926_rep-8 | 109 | 4.1 | 1 |  |
| AY642926 | 69261 | 69583 | AY642926_rep-9 | 322 | 3.9 | 1 |  |
| AY642926 | 74545 | 74638 | AY642926_rep-10 | 93 | 43.1 | 0 | SSR |
| AY642926 | 143551 | 143726 | AY642926_rep-11 | 175 | 6.2 | 17 |  |
| AY642926 | 143769 | 143989 | AY642926_rep-12 | 220 | 6.9 | 5 |  |
| AY643842S3 | 123451 | 123541 | AY643842S3_rep-1 | 90 | 1.7 | 4 |  |
| AY661558 | 30783 | 31012 | AY661558_rep-1 | 229 | 2.0 | 5 |  |
| AY661558 | 132411 | 132826 | AY661558_rep-2 | 415 | 42.5 | 30 |  |
| AY661558 | 132999 | 133133 | AY661558_rep-3 | 134 | 9.3 | 3 |  |
| AY661558 | 133174 | 133862 | AY661558_rep-4 | 688 | 19.2 | 14 |  |
| AY661558 | 133895 | 134833 | AY661558_rep-5 | 938 | 29.0 | 90 |  |
| AY661558 | 134875 | 135471 | AY661558_rep-6 | 596 | 51.6 | 23 |  |
| AY661558 | 152714 | 152988 | AY661558_rep-7 | 274 | 2.8 | 3 |  |
| AY661558 | 153040 | 153187 | AY661558_rep-8 | 147 | 4.0 | 1 |  |
| AY661558 | 184385 | 184474 | AY661558_rep-9 | 89 | 13.5 | 5 |  |
| AY661558 | 290338 | 290435 | AY661558_rep-10 | 97 | 1.7 | 4 |  |
| EF067844 | 6955 | 7072 | EF067844_rep-1 | 117 | 17.0 | 7 |  |
| EF067844 | 105741 | 105825 | EF067844_rep-2 | 84 | 19.3 | 4 |  |
| EF067844 | 259025 | 259163 | EF067844_rep-3 | 138 | 43.5 | 8 |  |
| EF067844 | 335857 | 336256 | EF067844_rep-4 | 399 | 5.6 | 1 |  |
| EF067844 | 336294 | 336399 | EF067844_rep-5 | 105 | 11.0 | 1 |  |
| EF067844 | 336439 | 336577 | EF067844_rep-6 | 138 | 5.5 | 1 |  |
| EF067844 | 336610 | 336707 | EF067844_rep-7 | 97 | 5.3 | 1 |  |
| EF067844 | 353574 | 353703 | EF067844_rep-8 | 129 | 69.2 | 4 |  |
| EF067844 | 353738 | 353937 | EF067844_rep-9 | 199 | 72.6 | 28 |  |
| EF067844 | 369445 | 369542 | EF067844_rep-10 | 97 | 107.3 | 95 |  |
| EF067844 | 427206 | 427297 | EF067844_rep-11 | 91 | 4.4 | 49 |  |
| EF067844 | 427376 | 427901 | EF067844_rep-12 | 525 | 3.6 | 22 |  |
| EF067844 | 428094 | 428212 | EF067844_rep-13 | 118 | 1.7 | 6 |  |
| EF067844 | 457963 | 458096 | EF067844_rep-14 | 133 | 3.5 | 30 |  |
